# Can Combination Therapy with Endothelin Receptor Antagonist and PDE5 Inhibitors Prevent Echocardiographic Findings Suspicious for Pulmonary Arterial Hypertension? Description of a Real-Life Case Series

**DOI:** 10.3390/diagnostics14141526

**Published:** 2024-07-15

**Authors:** Arianna Damiani, Gemma Lepri, Francesco Bonomi, Elisa Fiorentini, Silvia Peretti, Jelena Blagojevic, Silvia Bellando Randone, Serena Guiducci

**Affiliations:** Department of Clinical and Experimental Medicine, Division of Rheumatology, University of Florence, 50134 Florence, Italy; gemma.lepri@unifi.it (G.L.); francesco.bonomi@unifi.it (F.B.); elifiorentini93@gmail.com (E.F.); silvia.peretti@unifi.it (S.P.); jelena308@gmail.com (J.B.); silvia.bellandorandone@unifi.it (S.B.R.); serena.guiducci@unifi.it (S.G.)

**Keywords:** systemic sclerosis, pulmonary hypertension, phosphodiesterase-5 inhibitors, endothelin receptor antagonist

## Abstract

Objective: To retrospectively evaluate the incidence rate (IR) of elevated echocardiographic estimated systolic pulmonary artery pressure (sPAP), suspected for pulmonary hypertension (PH), in systemic sclerosis (SSc) patients after the introduction of a combination therapy with bosentan and sildenafil for treatment or prevention of digital ulcers. Methods: Patients attending the Scleroderma Unit of the Universital Hospital of Careggi from July 2010 to July 2023 were enrolled. Patients older than 18 years old with a history of digital ulcers, treated with bosentan and sildenafil in combination for at least 12 months, were included. Patients with a diagnosis of PH preceding the introduction of the therapy were excluded. Demographical data, disease duration, laboratoristic, and instrumental data (pulmonary function tests, echocardiographic estimation of sPAP, and ultrasonographic value of renal resistive index) were collected. The IR of echocardiographic signs suspected of pulmonary hypertension and their 95% confidence interval were calculated in events/1000 patients-years. Results: Thirty-five patients were enrolled; the mean disease duration was 12.82 years (SD 5.92). The mean duration of the combination treatment was 81.03 (SD 43.1.3) months, and the total at-risk time was 2674 months. Two patients (5.7%) presented echocardiographic signs of PH (sPAP 50 mmHg and 40 mmHg); the IR was calculated to be 9/1000 patients-years (95% CI 7.95–10.12). In one of the two patients, right heart catheterism (RHC) excluded PAH, while the other patient refused to undergo RHC, and PAH could not be confirmed/excluded. The stability of PFTs and echocardiographic sPAP was observed during the observation time. Conclusions: The results of this retrospective study suggest that combination therapy with endothelin receptor antagonists and phosphodiesterase-5 (PDE5) inhibitors could help in preventing PAH in SSc; prospective case–control studies on a larger population are needed to improve knowledge in this field.

## 1. Introduction

Systemic sclerosis (SSc) is a chronic connective tissue disease characterized by vasculopathy, inflammation with activation of the immune system, and the production of specific antibodies and fibrosis of the skin and internal organs [1]. Nowadays, early recognition of the disease and prompt treatment, along with a constant follow-up, permit to reduce SSc morbidity and mortality, but severe manifestations are still a challenge for rheumatologists involved in SSc management. Moreover, as the expectancy of life for SSc patients increases, new needs for treating a long-standing disease are emerging. Pulmonary arterial hypertension (PAH) is a serious complication of SSc vasculopathy, representing one of the main causes of SSc-related deaths together with interstitial lung disease (ILD) [2]. Pulmonary hypertension (PH) is a hemodynamic state defined by a mean pulmonary arterial pressure (mPAP) > 20 mmHg at rest at right heart catheterization (RHC) that still represents the gold standard diagnostic test. PH may be caused by different clinical conditions, often requiring a multidisciplinary approach, and it is clinically classified in five groups [3]: PAH (group 1), PH associated with left heart disease (group 2), PH associated with lung disease and/or hypoxia (group 3), PH associated with pulmonary artery obstructions (group 5), and PH with unclear and/or multifactorial mechanisms (group 6).

SSc patients may present different clinical conditions associated with PH; in fact, aside from PAH, patients can develop group 2 PH due to left heart dysfunction, group 3 PH because of ILD, and, less commonly, pulmonary veno-occlusive disease (group 1). In this context, a previous study reported 19% of PAH, 6% of group 2 PH, and 6% of group 3 PH among 466 SSc patients subjected to RHC [2]. Once PH is suspected, differentiating between these groups is mandatory to establish the prognosis and select the more appropriate treatment, and RHC is the only diagnostic tool that guarantees a definite diagnosis.

The prevalence of PAH within the SSc population is estimated between 6.4% and 9% [4], varying particularly according to the disease subset, and it seems to remain quite stable over time. The incidence of SSc-PAH in patients with limited cutaneous SSc and diffuse cutaneous SSc is estimated to be 1.25 and 0.4 cases per 100/patient-years, respectively [5]. During the last period, both innovation in the treatment and timely diagnosis of PAH improved its prognosis, but the mortality rate remains high, with a three-year survival rate of 62% [6], and an estimated 10-year survival rate of only 26.8% [7]. The prognosis of SSc patients with PAH is strongly linked to the early detection of PAH, and for this reason, the prompt identification of patients at major risk of developing this complication is mandatory. Major risk factors for PAH development include the positivity of anti-centromere antibodies (ACA), extensive telangiectasias, and longer disease duration [8]. In contrast, the protective factors for PAH have not yet been established, and this may be an interesting field for future research.

In addition, PAH in SSc seems to have a worse outcome than the idiopathic form, and now, no prevention pharmacological strategy has been identified to avoid this life-threatening condition [9]. Moreover, the response to specific PAH therapies is linked to the function class at the time of diagnosis, and for all these reasons, over the years, different screening processes have been proposed to detect this condition early [10]. Among these, the DETECT (detection of pulmonary arterial hypertension in SSc), the ASIG (Australian Scleroderma Interest Group), and the ITINER-Air (French multicenter transversal observational study) are composite algorithms commonly available in clinical practice [10].

Beyond these screening algorithms, echocardiographic parameters are widely used as screening tools for PAH, in particular systolic pulmonary arterial pressure (sPAP), which is also included in the French model. Other echocardiography parameters are evaluated in clinical practice, such as the tricuspid regurgitation velocity (TRV) and the right atrium area, which are both included in the DETECT algorithm. In addition to echocardiography, other examinations are routinely performed in SSc patients as screening tools for PAH, including pulmonary function tests (PFTs), with particular attention to lung diffusion for carbon oxide (DLCO) and biomarkers as the N-terminal pro-B-type natriuretic peptide (NT-proBNP). Among clinical manifestations, the presence of unexplained dyspnoea is an unspecific symptom that may indicate the presence of PAH and must lead to an instrumental evaluation. 

In this context, the recent ESC/ERS guidelines for the diagnosis and treatment of PH remarked on the importance of echocardiography as the first-line, non-invasive, diagnostic investigation in suspected PH, particularly assessing the TRV and the presence of other signs that are suggestive of PH.

Once SSc-related PAH is diagnosed, therapies are based on underlining pathogenetic pathways and patients’ risk assessments, including prostacyclin analogs, endothelin receptor antagonists (ERAs), phosphodiesterase isoenzyme 5 inhibitors (PDE5is), and guanylate cyclase stimulators (riociguat). According to the ESC guidelines, patients in the high-risk group should start PAH pharmacotherapy with a combination of triple therapy containing ERA, PDE5i, and parenteral prostacyclin. Patients with low- or intermediate-risk conditions should start with dual combination therapy containing ERA and PDE5i or riociguat. 

Among the vascular therapeutic options for SSc, ERAs account for both vasodilating and anti-fibrotic properties, and bosentan, a dual ERA, has proven effective in healing active ulcers [11] and preventing their recurrence. PDE5s, such as sildenafil, promote vascular smooth muscle relaxation and vasodilatation [12]. For this reason, both bosentan and sildenafil are widely used in patients with PAH, and they are also indicated in treating digital ulcers (DUs) and Raynaud’s phenomenon, respectively.

The aim of this retrospective study is to evaluate the incidence of an elevation of echocardiographic esteem of the sPAP over 40 mmHg, suspected for PH, in SSc patients treated with bosentan and sildenafil for peripheral vasculopathy (active DUs or prevention of their recurrence).

## 2. Materials and Methods

SSc patients attending the Scleroderma Unit of the Universital Hospital of Careggi from July 2010 to July 2023 were enrolled in a retrospective observational study. Inclusion criteria were as follows: aged ≥ 18 years old, to be classified as SSc according to ACR/EULAR 2013 classification criteria [13], a history of digital ulcers (DUs), to be treated with bosentan and sildenafil in combination for at least 12 months, and to have echocardiographic evaluation at baseline (time of bosentan and sildenafil introduction) and at the end of the observation period. Bosentan was administered at a dosage of 125 BID die; sildenafil was administered at a dosage of 20 mg TID die. Patients with a diagnosis of PAH or PH at RHC preceding the introduction of the therapy were excluded from the study. For each patient, demographical data, disease features (SSc-specific antibodies, renal arterial resistive index assessed by ultrasound examination), and data on combination therapies were collected at baseline. In addition, echocardiographic parameters (sPAP) and PFT values (forced vital capacity (FVC) and DLCO expressed as percentages of theoretical measures) were collected for all patients at baseline and at the end of the observation period. We considered sPAP > 40 mmHg the echocardiographic sign to suspect the presence of PH.

Statistical analysis was made through the software R 3.5.2 GUI 1.70 El Capitan build (7612). Categorical variables were described using frequencies and percentages; numerical variables were described by mean and standard deviation. The incidence rate of echocardiographic signs suspected of pulmonary hypertension (sPAP > 40 mmHg) in the study population and its 95% confidence interval were calculated in events/1000 patients-years. When available, the data of RHC were recorded, defining a case of PAH as a mean pulmonary arterial pressure (mPAP) of 20 mmHg, a pulmonary capillary wedge pressure (PCPw) of 15 mmHg, and pulmonary vascular resistance > 3 Wood units [14].

Baseline and follow-up PFTs and sPAP were compared using a paired T test, with significance levels fixed at 5%.

The study was approved by the Ethical Committee of Florence: 37/2008. All patients gave their written informed consent.

## 3. Results

A total of 35 patients, with a diagnosis of SSc and past or active DUs, received a vasoactive treatment with bosentan and sildenafil for at least 12 months and were enrolled in the study. Among them, 31 (88.6%) were female; the mean age was 59.29 years old (SD 14.37), and the mean disease duration was 12.82 years (SD 5.92). A total of 14 patients (46.7%) were positive for anti-topoisomerase I (anti-topo I) antibodies, while 11 (36.7%) were ACA-positive; none had anti-RNA polymerase III antibodies. Eight (22.9%) patients had lung involvement in the presence of interstitial lung disease. 

The mean duration of the treatment was 81.03 (SD 43.1.3) months, corresponding to the mean follow-up of the included patients, and the total at-risk time was 2674 months. 

In Table 1, the population characteristics and baseline laboratoristic and instrumental data (NT-proBNP, PFTs parameters, echocardiographic sPAP, and RI values) are reported. As described, the mean basal sPAP was within the range of normality.

Among all of the patients, only two patients (4.2%), at the end of follow-up, presented echocardiographic signs of pulmonary hypertension (sPAP ≥ 40 mmHg), with an sPAP of 50 mmHg and 40 mmHg, respectively. The incidence rate of the echocardiographic sign of PH was calculated to be 9/1000 patients-years (95% CI 7.95–10.12).

RHC was proposed to both patients with echocardiographic signs of pulmonary hypertension, but one refused this examination. In the other patient, RHC showed hemodynamic findings that were compatible with mild post-capillary PH, and PAH was ruled out. As reported in Table 2, a stability in PFT values and echocardiographic sPAP was observed from baseline to the end of follow-up in the studied population. No scleroderma renal crisis was recorded during the observation period, and neither did DUs relapse. ILD was present in the patient with RHC-diagnosed post-capillary hypertension, while the other patient with elevated sPAP did not present fibrotic lung involvement. In both patients, other causes of PAP elevation (i.e., pulmonary embolism, cardiopathies) were ruled out. 

## 4. Discussion

PAH is caused by a proliferative remodeling of small pulmonary arteries, representing a fearful manifestation of SSc, and according to the 2022 ESC/ERS guidelines, it is hemodynamically defined as a pre-capillary PH (in the absence of other causes of pre-capillary PH) with a mPAP > 20 mmHg, a PAWP (pulmonary arterial wedge pressure) ≤ 15 mmHg and a PVR (pulmonary vascular resistance) > 2 WU at RHC [3]. The prevalence of PAH within the SSc population is highly variable, particularly according to the disease subset. Major risk factors for PAH development include the positivity of anti-centromere antibodies, longer disease duration, and the presence of other signs of vasculopathy, such as extensive cutaneous telangiectasia [15], digital ulcers [16], and digital pitting scars [17]. PAH remains a major cause of death in SSc patients; however, over the last few years, the management of this complication has certainly improved, thanks to the use of new drugs. In this context, the AMBITION trial [18] showed the benefits of the early introduction of combination therapy with once-daily ambrisentan and tadalafil, as compared with monotherapy with either of these agents, with primary outcomes such as a composite end point of death, hospitalization for worsening PAH, disease progression, or unsatisfactory long-term clinical responses. However, to our knowledge, few studies have evaluated the incidence of new PAH cases in SSc patients treated with vasoactive drugs because of the presence of DUs. Castellvì et al. [19] conducted a retrospective case–control study in 237 SSc patients with a previous history of DUs and compared the occurrence of new PH echocardiographic diagnosis in patients treated (n = 59) and not-treated (n = 163) with bosentan for at least one month. The median treatment duration was 34 (CI 95% 5–59) months. During the follow-up, 13.8% of the treated patients with bosentan and 23.7% of the not-treated developed PH based on echocardiography definition (OR = 0.52; CI 95% = 0.22–1.19), without significant differences between the two groups. However, the authors performed a multivariate analysis, finding that patients without bosentan had a baseline higher risk of developing PH (3.91 (CI 95%:1.3–11.6; *p* < 0.02), so bosentan could have been protective against PH in this group. Caramaschi et al. [20] evaluated the incidence of PH in 81 patients treated with cyclic iloprost infusion for severe Raynaud phenomena and/or DUs. At the end of the follow-up (61.3 ± 29.3) period, nine patients presented with echocardiographic signs of PH. One of them underwent RHC, which excluded PAH. Even if the incidence rate of PH in this study was in line with the one reported in the literature concerning larger cohorts, the authors stated that none of the included patients developed severe isolated pulmonary hypertension. In 2021, Pestaña-Fernández et al. [21] analyzed 544 patients with SSc and a history of DUs. Out of them, 221 were treated with ERA and/or PDE5is, while the other 323 did not receive vasoactive therapy. The incidence rate of PAH diagnosed on the basis of RHC resulted in 7.7 (95% CI 4.7, 12.0) per 1000 person-years in the treated group compared with 7.8 (95% CI 5.2, 11.3) per 1000-person-years (*p* value of 0.988) in the no-treatment group. The incidence rate difference between groups did not achieve statistical significance [0.1 (4.8, 4.69); *p* 0.988]. The authors stated that patients treated with ERA or PDE5is could show more severe vascular involvement. Moreover, a longer time period between the DUs’ presentation and scleroderma renal crisis development was observed so that the vasoactive drug could exert a protective role. However, the authors did not perform a subanalysis to compare patients in monotherapy and patients in combination therapy with ERA and PDE5is. 

Our study evaluated the presence of echocardiographic elevation of sPAP in patients treated with combination therapy with bosentan and sildenafil for peripheral vasculopathy, revealing that only 2/47 patients showed an increase in sPAP at the end of the observation period (56 and 130 months). One patient was subjected to RHC, and a mild post-capillary PH was diagnosed. Unfortunately, one of the two patients with increased sPAP at follow-up refused the RHC, which may not have confirmed the presence of a PAH, reinforcing the hypothesis that the combo therapy could prevent PAH development. Overall, our data reported a stabilization of echocardiography sPAP in patients with DUs treated with bosentan and sildenafil, suggesting a potential preventive role of this combination therapy in the development of instrumental alterations, which is suggestive of PH in this group of patients. As reported above, echocardiography is a first-line, non-invasive, diagnostic investigation in suspected PH. Therefore, from a clinical point of view, the data from our study translate into a low number of patient candidates for RHC, which represents a more (even if minimally) invasive examination. 

Our study presents some limitations: First, the absence of a control group is a major limitation; however, this is justified by our clinical practice. In fact, in our center, all patients with DUs or with a history of DUs are candidates for combination therapy with a vasoactive treatment (bosentan) and a vasodilator (sildenafil or iloprost) if no contraindications are present. Therefore, the control population (patients not treated with bosentan and sildenafil) at our disposal was represented by SSc subjects without DUs that are known themselves to represent a severe sign of peripheral vascular disease associated with PAH. In addition, another major limitation is that the only echocardiographic parameter available for all patients at baseline and follow-up was represented by sPAP, while the data on TRV and the right atrium area were available for a few patients, not allowing a statistical analysis of these values and a complete evaluation according to 2022 ESC/ERS guidelines. Other limitations of our study include the small sample size, its retrospective nature, and the relatively short follow-up that prevented us from performing a definitive comparison with the PAH epidemiological data. Anyway, in our population, the low incidence of increased values of sPAP, along with the stability of PFT parameters, enable us to be optimistic toward the protective role of the combination therapy against SSc PAH, indicating that its routine administration in patients with severe Raynaud phenomenon/DUs could help lower the incidence and mortality rates of this challenging SSc complication.

In conclusion, the results of this retrospective study suggest the possible role of combination therapy with bosentan and sildenafil in preventing PH in SSc patients with DUs. The confirmation of the preventive role of a combination therapy of endothelin antagonist receptors and phosphodiesterase-5 inhibitors against PAH development in SSc patients now needs further prospective studies in a larger cohort of patients, considering potential confounders such as the presence of peripheral vasculopathy (DUs or teleangectasias). Moreover, further studies should overcome the limitations of the present study, analyzing all echocardiographic signs of PH according to the 2022 ESC/ERS guidelines.

## Figures and Tables

**Table 1:** Baseline clinical, laboratoristic, and instrumental data.

	n° (%)
Females	31 (88.6)
Age, mean (SD)	59.20 (14.37)
Disease duration, mean (SD)	12.82 (5.92)
Autoantibodies, n° (%)	
ACA	11 (36.7)
anti-topo I	14 (46.7)
Anti-RNP	1 (3.3)
Anti Pm/Scl.	1 (3.3)
Anti Ro52	2 (6.7)
Anti Sm	1 (3.3)
Presence of ILD, n° (%)	8 (22.9)
FVC (%), mean (SD)	98.52 (14.43)
DLCO (%), mean (SD)	67.07 (16.99)
sPAP (mmHg), mean (SD)	28.67 (5.37)
NT-proBNP (pg/mL), mean (SD)	438.52 (596.8)
Renal resistive index	70.78 (4.49)

ACA: anti-centromer antibodies; ILD: interstitial lung disease; FVC: forced vital capacity; DLCO: diffusion for carbon oxide; sPAP: systolic pulmonary artery pressure.

**Table 2:** Comparison between functional and echocardiographic baseline and follow-up values.

	BL	FU	*p* Value
FVC (%), mean (SD)	98.52 (14.43)	8.09 (24.27)	0.831
DLCO (%), mean (SD)	67.07 (16.99)	68.72 (19.78))	0.972
sPAP (mmHg), media (SD)	28.67 (5.37)	30.17 (7.57)	0.109

BL: baseline; FU: follow-up; FVC forced vital capacity; DLCO: diffusion for carbon oxide; sPAP: systolic pulmonary artery pressure.

## Data Availability

The raw data supporting the conclusions of this article will be made available by the authors on request.

## References

[1] Varga J., Trojanowska M., Kuwana M. (2017). Pathogenesis of systemic sclerosis: Recent insights of molecular and cellular mechanisms and therapeutic opportunities. J. Scleroderma Relat. Disord..

[2] Steen V.D., Medsger T.A. (2007). Changes in causes of death in systemic sclerosis, 1972–2002. Ann. Rheum. Dis..

[3] Humbert M., Kovacs G., Hoeper M.M., Badagliacca R., Berger R.M., Brida M., Carlsen J., Coats A.J.S., Escribano-Subias P., Ferrari P. (2022). 2022 ESC/ERS Guidelines for the diagnosis and treatment of pulmonary hypertension: Developed by the task force for the diagnosis and treatment of pulmonary hypertension of the European Society of Cardiology (ESC) and the European Respiratory Society (ERS). Endorsed by the International Society for Heart and Lung Transplantation (ISHLT) and the European Reference Network on rare respiratory diseases (ERN-LUNG). Eur. Heart J..

[4] Rubio-Rivas M., Homs N.A., Cuartero D., Corbella X. (2021). The prevalence and incidence rate of pulmonary arterial hypertension in systemic sclerosis: Systematic review and meta-analysis. Autoimmun. Rev..

[5] Haque A., Kiely D.G., Kovacs G., Thompson A.A.R., Condliffe R. (2021). Pulmonary hypertension phenotypes in patients with systemic sclerosis. Eur. Respir. Rev..

[6] Morrisroe K., Stevens W., Huq M., Prior D., Sahhar J., Ngian G.-S., Celermajer D., Zochling J., Proudman S., the Australian Scleroderma Interest Group (ASIG) (2017). Australian Scleroderma Interest Group (ASIG). Survival and quality of life in incident systemic sclerosis-related pulmonary arterial hypertension. Arthritis Res. Ther..

[7] Chen X., Quan R., Qian Y., Yang Z., Yu Z., Zhang C., Yang Y., Zhang G., Shen J., Wang Q. (2023). 10-year survival of pulmonary arterial hypertension associated with connective tissue disease: Insights from a multicenter PAH registry. Rheumatology.

[8] Shah A.A., Wigley F.M., Hummers L.K. (2010). Telangiectases in scleroderma: A potential clinical marker of pulmonary arterial hypertension. J. Rheumatol..

[9] Launay D., Sitbon O., Hachulla E., Mouthon L., Gressin V., Rottat L., Clerson P., Cordier J.-F., Simonneau G., Humbert M. (2013). Survival in systemic sclerosis-associated pulmonary arterial hypertension in the modern management era. Ann. Rheum. Dis..

[10] Bruni C., De Luca G., Lazzaroni M.G., Zanatta E., Lepri G., Airò P., Dagna L., Doria A., Matucci-Cerinic M. (2020). Screening for pulmonary arterial hypertension in systemic sclerosis: A systematic literature review. Eur. J. Intern. Med..

[11] Hughes M., Bruni C., Ruaro B., Confalonieri M., Matucci-Cerinic M., Bellando-Randone S. (2021). Digital Ulcers in Systemic Sclerosis. Presse Méd..

[12] Heresi G.A., Minai O.A. (2008). Bosentan in systemic sclerosis. Drugs Today.

[13] Van den Hoogen F., Khanna D., Fransen J., Johnson S.R., Baron M., Tyndall A., Matucci-Cerinic M., Naden R.P., Medsger T.A., Carreira P.E. (2013). 2013 classification criteria for systemic sclerosis: An American college of rheumatology/European league against rheumatism collaborative initiative. Ann. Rheum. Dis..

[14] Galiè N., Humbert M., Vachiery J.L., Gibbs S., Lang I., Torbicki A., Hoeper M. (2016). 2015 ESC/ERS Guidelines for the diagnosis and treatment of pulmonary hypertension: The Joint Task Force for the Diagnosis and Treatment of Pulmonary Hypertension of the European Society of Cardiology (ESC) and the European Respiratory Society (ERS): Endorsed by: Association for European Paediatric and Congenital Cardiology (AEPC), International Society for Heart and Lung Transplantation (ISHLT). Eur. Heart J..

[15] Hurabielle C., Avouac J., Lepri G., de Risi T., Kahan A., Allanore Y. (2016). Skin Telangiectasia and the Identification of a Subset of Systemic Sclerosis Patients With Severe Vascular Disease. Arthritis Care Res..

[16] Mihai C., Landewé R., van der Heijde D., Walker U.A., Constantin P.I., Gherghe A.M., Ionescu R., Rednic S., Allanore Y., Avouac J. (2016). Digital ulcers predict a worse disease course in patients with systemic sclerosis. Ann. Rheum. Dis..

[17] Hughes M., Heal C., Henes J., Balbir-Gurman A., Distler J.H.W., Airò P., Müller-Ladner U., Hunzelmann N., Kerzberg E., Rudnicka L. (2022). Digital pitting scars are associated with a severe disease course and death in systemic sclerosis: A study from the EUSTAR cohort. Rheumatology.

[18] Galiè N., Barberà J.A., Frost A.E., Ghofrani H.-A., Hoeper M.M., McLaughlin V.V., Peacock A.J., Simonneau G., Vachiery J.-L., Grünig E. (2015). AMBITION Investigators. Initial Use of Ambrisentan plus Tadalafil in Pulmonary Arterial Hypertension. N. Engl. J. Med..

[19] Castellví I., Simeón C.P., Sarmiento M., Casademont J., Corominas H., Fonollosa V. (2020). Effect of bosentan in pulmonary hypertension development in systemic sclerosis patients with digital ulcers. PLoS ONE.

[20] Caramaschi P., Volpe A., Tinazzi I., Bambara L.M., Carletto A., Biasi D. (2006). Does cyclically iloprost infusion prevent severe isolated pulmonary hypertension in systemic sclerosis? Preliminary results. Rheumatol. Int..

[21] Pestaña-Fernández M., Rubio-Rivas M., Tolosa-Vilella C., Guillén-Del-Castillo A., Colunga-Argüelles D., Argibay A., Marí-Alfonso B., Marín-Ballvé A., Pla-Salas X., Chamorro A.-J. (2021). The incidence rate of pulmonary arterial hypertension and scleroderma renal crisis in systemic sclerosis patients with digital ulcers on endothelin antagonist receptors (ERAs) and phosphodiesterase-5 inhibitors (PDE5i). Rheumatology.

